# Prevalence of Metabolic Syndrome and Its Parameters and Their Correlations With Psoriasis Duration, Severity, and Sleep Quality In Psoriasis Patients: A Cross-Sectional Study

**DOI:** 10.5826/dpc.1103a49

**Published:** 2021-05-20

**Authors:** Betul Tas, Vasfiye Kabeloglu

**Affiliations:** 1 Department of Dermatology and Venereology, University of Health Sciences, Istanbul Bagcilar Research and Training Hospital, Istanbul, Turkey; 2 Department of Neurology, University of Health Sciences, Bakirkoy Prof. Dr. Mazhar Osman Research and Training Hospital, Istanbul, Turkey

**Keywords:** psoriasis, metabolic syndrome, sleep quality, comorbidity

## Abstract

**Background:**

Psoriasis is an inflammatory skin disease that may lead to comorbidities, including metabolic syndrome (MS).

**Objective:**

We determined the prevalence of MS and its correlation with psoriasis duration, severity, and sleep quality in psoriasis patients.

**Methods:**

A total of 112 subjects with chronic plaque psoriasis were studied. Demographics, MS parameters, disease duration, severity, and sleep quality were examined. The Psoriasis Area and Severity Index (PASI) and the Pittsburgh Sleep Quality Index (PSQI) were used to assess psoriasis severity and sleep quality, respectively. Presence of MS and its correlations with psoriasis duration, severity and sleep quality were investigated.

**Results:**

Of 112 patients, 76 (67.8%) were diagnosed with MS. Of all patients, 74.1% had a high PASI, and 84.8% had a high PSQI. The mean values of psoriasis duration, body mass index, waist circumference, fasting glucose, HOMA-IR, triglyceride levels, blood pressure, PSQI, sleep latency, and daytime sleep dysfunction were significantly higher in the MS group than non-MS group, whereas the mean HDL level was lower. The prevalences of MS, high fasting glucose, and low HDL were significantly higher among female, but not male, patients with severe psoriasis (PASI >10) than those without severe psoriasis. Disease duration, high body mass index, waist circumference, blood pressure, fasting glucose, HOMA-IR, triglyceride levels, low HDL, and poor sleep quality were significantly correlated with the presence of MS. However, only waist circumference, fasting glucose, blood pressure, and low HDL were predictive of the development of MS.

**Conclusions:**

MS is common among psoriasis patients, and especially in females with advanced psoriasis, high fasting glucose, and low HDL levels. Besides diagnostic criteria of MS, a long duration of psoriasis, poor sleep quality and high-HOMA-IR correlate with the development of MS. High fasting glucose and low HDL levels may facilitate MS development in association with psoriasis severity in females.

## Introduction

Psoriasis is an immune-mediated, inflammatory skin disease. Because of the increasing rate of coexistence with many co morbidities, psoriasis is starting to be considered a multisystem disease. Many comorbidities have been described, such as arthritis, obesity, hypertension, dyslipidemia, diabetes mellitus, cardiovascular diseases (CVDs), and sleep disorders [1,2]. Various factors play roles in the sharing of pathogenetic mechanisms between psoriasis and these comorbidities. Common pathways and cytokine profiles are used to explain this interaction. Thanks to these relationships, psoriasis is beginning to be associated with metabolic syndrome (MS) [3]. On the other hand, MS itself is a multisystem disease that is associated with disorders such as hypertension, dyslipidemia, high blood glucose, and CVDs.

Psoriasis and obesity are chronic inflammatory diseases, and a strong correlation has been reported between these two disorders. Abdominal obesity is considered the most important parameter in the development of MS in psoriasis patients [4]. Adipose tissue secretes cytokines like tumor necrosis factor alpha (TNF-alpha), which induces the production of free fatty acids, decreases adiponectin synthesis, disrupts insulin signaling, and ultimately leads to insulin resistance [5]. Levels of TNF-alpha has been found to be significantly higher in the skin of psoriasis patients than unaffected people [6,7]. Thus, it is suggested that obesity plays an important role in increasing the risk of development of MS, diabetes mellitus and CVDs in psoriasis patients [8,9]. Psoriasis may also lead to poor sleep quality, because it causes itchiness and sleep disturbances [10,11]. Thus, we investigated the presence of MS and MS parameters and their correlations with psoriasis duration, severity, and sleep quality, and also detected predictive factors in the development of MS in psoriasis patients.

## Patients and Methods

This was a cross-sectional, observational study. The study protocol was approved by the Ethics Committee of University of Health Sciences, Istanbul Bagcilar Research and Training Hospital (approval code, 2018.11.1.08.108), and written informed consent was obtained from all study subjects. Study participants were recruited from patients who attended our dermatology clinics between January and July 2019. The study was conducted in accordance with the World Medical Association’s Declaration of Helsinki, 2013.

Inclusion criteria were: ≥18 years of age, clinical and histopathological diagnosis of psoriasis, proficiency in communication, enough understanding of questionnaires, having no other dermatological disorders, and absence of a previous MS-diagnosis. Exclusion criteria were: cognitive impairment, having other dermatological, neurological, ear-nose-throat disorders, or having an acute upper-respiratory-tract infection that could cause poor sleep quality. Patients were recruited until the number enrolled reached a previously determined sample size (it was detected using a sample calculation with a single group proportional data size). Referencing the average number of patients who were admitted to our clinics in the determined period, the sample size was determined to be 99 with a 95% level of confidence and a 5% of margin of error. However, approximately 10% more subjects were enrolled, in case any of the enrolled subjects dropped out of the study.

### Data Collection and Analyses

The study subjects’ age, gender, disease duration, smoking habit, alcohol consumption habit were recorded, as were previously diagnosed coexistent chronic diseases, namely diabetes mellitus, hypertension, lung disease, chronic kidney disease, cerebrovascular disease, and thyroid disease. Psoriasis severity was evaluated using the Psoriasis Area and Severity Index (PASI) [12]. This index combines a score for severity (erythema, induration and desquamation, grades of 0–4) and the percentage of affected areas (head, arms, trunk and legs, from 0% to 100%). Minimum and maximum scores are 0 and 72, respectively. Values higher than 10 indicate moderate-severe psoriasis.

Sleep quality was evaluated with the Pittsburgh Sleep Quality Index (PSQI), which measures sleep quality over the previous month, and includes 19 questions to be answered by patients and 5 questions rated by their bed or room partner (if one exists). The questions form the component scores, each of which has a range of 0–3 points. A score of 0 indicates no difficulty in sleep, while a score of 3 indicates severe difficulty. The 7 sub-component scores, which indicate subjective sleep quality (PSSQ), sleep latency (PSL), sleep duration (PSDu), habitual sleep efficiency (PHSE), sleep disturbances (PSDi), use of sleeping medication (PUSM), and daytime sleep dysfunction (PDSD), add up to a total score (0–21 points). A total score ≥5 indicates poor sleep quality [13]. The PSQI was adapted to the Turkish population by Agargun et al [14].

The National Cholesterol Education Program Adult Treatment Panel III (ATP III) criteria were used to investigate MS parameters [15]. First, the anthropometric parameters of weight, body mass index (BMI), and waist circumference, and systolic and diastolic blood pressures (SBP, DBP) were measured [14]. Subjects who had ≥3 of the following parameters were diagnosed with MS: fasting glucose ≥110 mg/dl; fasting triglyceride ≥150 mg/dl; HDL<50 mg/dl for women and<40 mg/dl for men; waist circumference>88cm for women and >102 cm for men; and SBP ≥130 mmHg or DBP ≥85 mmHg [15]. BMI was calculated as weight (kilograms) divided by height (square meters), and was evaluated as non-obese (<30) and obese (≥30) [16]. Waist circumference was measured at the midpoint between the twelfth rib and the iliac crest [17]. SBP and DBP were measured following the JNC-7 guidelines [18]. Plasma fasting glucose, HDL and triglyceride levels were analyzed by automated enzyme immunoassay and serum insulin levels were measured using an electrochemiluminescence immunoassay (Cobras 6000-601 automated analyzer, Roche Diagnostics). Insulin resistance was detected by calculating the homeostatic model assessment-insulin resistance (HOMA-IR) index as follows: HOMA-IR=fasting insulin (mIU/l)×fasting glucose (mg/dl)/405 [19]. Values of HOMA-IR were considered normal if <2.5 and high if ≥2.5 [20].

### Statistical Analyses

NCSS (Number Cruncher Statistical System, 2007) software was used. Standard descriptive statistics were expressed as mean and standard deviation (SD) or number and percentage. The normality assumptions were checked using the Kolmogorov-Smirnov test. Independent-samples t-test was used to compare quantitative data when their distribution was normal, whereas the Mann-Whitney U test was used to compare means when the distribution was not normal. Qualitative data was compared with Pearson’s chi-square test. Spearman’s rho test was used for the correlation of dependent and independent variables which were distributed asymmetrically, A P value <.05 was considered significant. Strength of correlations was determined according to rho (r) values, as follows: r< 0.2 (very weak, no correlation), r=0.2–0.4 (weak), r=0.4–0.6 (moderate), r=0.6–0.8 (strong), and r> 0.8 (very strong). Predictivity of determinant factors was detected with logistic regression analysis. Results were determined with 95% confidence.

## Results

A total of 112 patients with psoriasis (53 men and 59 women) were included in the study (Table 1). Their mean age was 46.5 years (SD = 13.2 years; range, 21–73 years). Overall, 50% of them was obese and 78.6% had high waist circumference; 42.0% had high fasting glucose, 83.0% had high triglyceride, and 61.6% had low HDL levels. Hypertension and high HOMA-IR were detected in 23.2% and 63.4% of cases, respectively. PASI values were high in 74.1% of subjects, whereas 84.8% had high PSQI values. The most detected comorbidity was hypertension (23.2%). Alcohol consumption was reported by 15.2%, whereas 38.4% were smokers.

MS was diagnosed in 76 (67.8%) of the 112 patients. Proportionately more women had MS, but this association was not significant (Table 2). More patients in the MS group than non-The MS group had high values of waist circumference, BMI, triglyceride levels, fasting glucose, blood pressure (each P = .0001) and HOMA-IR (P= .014), and low values of HDL (P= .0001). More patients in the MS group had PSQI scores ≥5(P = .046), while similar percentages of patients in both groups had PASI scores >10.

Mean values of age, disease duration, MS parameters, PASI, PSQI and its subgroups are compared in Table 3. The means of disease duration, BMI, waist circumference, fasting glucose, HOMA-IR, triglyceride levels, PSQI, PSL, and PDSD were significantly higher, and HDL values were significantly lower in the MS group.

Then, for all 112 patients, MS parameters were compared in subgroups according to PASI (≤10 vs. >10) and gender (Table 4). High fasting glucose, low HDL levels, and a diagnosis of MS were significantly more frequent only in females with high PASI (P =.003, P = .027, P = .031, respectively).

Correlations between diagnosis of MS and clinical characteristics such as psoriasis duration, PASI and PSQI are shown in Table 5. Positive correlations were detected between MS and disease duration, BMI, waist circumference, high blood pressure, fasting glucose, HOMA-IR, triglyceride levels and PSQI, whereas HDL values were negatively correlated with it.

The impacts of the correlative parameters on MS development are shown in Table 6. Only waist circumference, fasting glucose, high blood pressure, and low HDL had predictive effects.

## Discussion

Psoriasis is a chronic inflammatory skin disease that affects 2%–4% of the general population [21,22]. The prevalence of MS has been reported to be 35% in the United States [21]. In the Turkish population, it has been reported to be 34.9% (25.2% in men and 40.1% in women) by Gündogan et al [23] and 33.9% by Gemalmaz et al [24]. Although there was a female predominance (52.7%) in our study, the prevalence of MS in men and women was similar (P =.230). Although the exact relationship is not fully understood, it is well known that psoriasis patients have a higher prevalence of MS, which changes with a person’s genetic susceptibility, aging, dietary habits, and sedentary behaviors [25]. In a comprehensive meta-analysis, the prevalence of MS among people with psoriasis was in the range of 20%–50%, in which adjusted ORs were reported as 1.22, 1.56, and 1.98 for mild, moderate and severe psoriasis, respectively [26]. Another meta-analysis of 12 studies found that the prevalence of MS was 2.2-times higher in psoriasis patients than in the general population. It was reported as 49.4% by Ferdinando et al [25], which was 1.8-times higher (35.0%) than in the control group. In another meta-analysis of 35 studies from 20 countries, the pooled OR for MS in psoriasis patients was 2.14 compared to the general population [21]. Additionally, ORs have been reported to be 2.94 and 3.65 in different provinces of Turkey, by Zindanci et al [27] and Tasliyurt et al [28]. Adisen et al found that the prevalence was 12.6% in 563 psoriasis patients [29], whereas Baeta et al showed that it was 44.9% in 190 psoriasis patients [30].

In our study, 67.8% of patients had MS (72.8% in females and 62.2% in males). Although the age range, mean age, and gender distribution of our study were similar to those in the aforementioned studies, they were still higher than in those studies. However, another study from Turkey indicated that MS prevalence was 43.5% in females and 41.4% in males in 2010; this study predicted that the prevalence of MS will increase in the coming years [29]. Considering this information [21,23,31] and due to the fact that MS is more common in psoriasis patients [25, 26, 29, 30, 31], our results are in line with the expectations mentioned. The role of age and gender on MS development in psoriasis patients varied in different studies. Kim et al [32] and Altunay et al [3] showed that the presence of MS was significantly higher in older psoriasis patients, and the prevalence was insignificantly higher in males. Adisen et al [29] also stated that it did not vary by gender but was higher in older people [29]. We did not find a significant difference according to either age or gender.

The exact mechanism for the link between psoriasis and MS is uncertain. However, common pathways through Th1/Th17 cells, and common effects of pro-inflammatory cytokines on adipocytes, glucose regulation, lipid status, and endothelial function are used to try to explain it [21, 25]. Psoriasis and MS can develop in a person independently of each other. However, it is suggested that the existence of one of them can facilitate the development of the other, because of a shared immunopathogenesis involving chronic inflammation mediated by cytokines such as IFN-gamma, IL-17, IL-23, and TNF-alpha. Similarly, insulin-like growth factor is a shared mediator for the development of diabetes mellitus, hyperlipidemia and keratinocyte proliferation in psoriatic skin [21]. It is also suggested that obesity in psoriasis patients plays a key role in the increased risk for MS development [8,9]. Common outcomes of both psoriasis and MS are abdominal obesity, hypertension, dyslipidemia, and insulin resistance [21].

Although the relationship between psoriasis severity and MS has been reported differently in the literature [33], MS prevalence usually increases with psoriasis severity, when MS components are considered individually [21]. However, Adisen et al [29]and Altunay et al [3] found that mean scores of PASI were not different between patients with and without MS, whereas Itani et al [33] reported that the mean PASI score was greater in psoriasis patients with than without MS. We did not find any difference between groups according to PASI. However, although the prevalences of MS were not different by gender, the presence of MS was significantly higher in females with high PASI than in males. Moreover, when considering different PASI groups, only fasting glucose and HDL levels significantly differed between the genders. Thus, higher rates of MS in women with severe psoriasis might be related to their high fasting glucose and low HDL values. On the other hand, it has been stated that MS development may be affected by psoriasis duration. Altunay et al’s study showed that there was no relationship between disease duration and the presence of MS [3], whereas Itani et al [33]and Adisen et al [29] reported that MS was significantly higher in psoriasis patients with longer than shorter duration, as in our study. Considering the different MS parameters, we found that the means of BMI, waist circumference, fasting glucose, triglyceride, and blood pressure were significantly higher in the MS group, whereas HDL levels were significantly lower. So, our results support the understanding that psoriasis patients are at risk of having all MS parameters.

Additionally, we assessed the prevalence of insulin resistance in our-study subjects. Polic et al [34] found a correlation between high HOMA-IR levels and a diagnosis of MS in psoriasis patients, and HOMA-IR was significantly higher in patients with severe psoriasis. Similarly, we detected significantly higher HOMA-IR levels in the MS group, but HOMA-IR was not related to psoriasis severity. Moreover, because of the stronger correlation of MS with fasting glucose than with HOMA-IR, the former may have more importance on the development of MS.

Alcohol use and a smoking habit may trigger or worsen psoriasis [29] and cardiovascular, metabolic and hepatic complications in MS [31]. Smoking may affect clinical variability or course of psoriasis because of causes or increases in oxidative stress, functional morphological changes in polymorphonuclear leukocytes, and release of chemotactic factors and cytokines like interleukins, TNF-alpha, and transforming growth factor beta. It is also suggested that alcohol makes psoriasis worse by affecting lymphocyte transformation. Rates of smoking and alcohol use have been reported as 20%–65% and 3.3%–24% in psoriasis patients, respectively [29]. This study found that these prevalences were 38.39% and 15.8%, which are consistent with the literature. However, both habits did not show any correlation with the presence of MS. On the other hand, sleep disturbances have been reported in approximately 50%–60% of psoriasis patients [35]. Hawro et al reported that 39% of psoriasis patients had overall poor sleep quality, especially daytime sleepiness and awakenings during sleep [36]. Disturbances in sleep quality have usually been associated with itchiness and low quality of life in psoriasis patients [37, 38]. Melikoğlu [39]and Jensen et al [40] reported that psoriasis patients had significantly poorer overall sleep quality than controls, at the rates of 60% and 53.9%, respectively, whereas Shutty et al [41] reported that poor sleep quality in psoriasis patients was 4.3-times higher than in healthy controls. However, Stinco et al [42] did not find any difference in sleep quality between psoriasis patients and healthy controls. In our study, 84.8% of patients had PSQI values ≥5, indicating poor sleep quality. This result agrees with the literature, but the rate is higher. Additionally, the PSQI mean was significantly higher in the MS group than non-MS group. Sleep latency and daytime sleep dysfunction were significantly higher in the MS group; these results are similar to those of Hawro et al [36]. Moreover, it is a well-known fact that the prevalence of respiratory difficulties such as obstructive sleep apnea is higher in obese patients, which lead to sleep disorders, and there is high prevalence of obesity in patients with psoriasis [11]. Because most of our MS subjects were obese, and there was no relation between the presence of MS and psoriasis severity in the whole group, their poor sleep quality might be more related to their obesity than their psoriasis severity. However, MS prevalence was significantly higher in females with severe psoriasis, compared to males. Because the significantly higher fasting glucose and lower HDL were also detected only in females with severe psoriasis, these abnormalities might be more related to the development of MS in females than in males. Considering the correlations between the presence of MS and study parameters, significant positives were detected between the presence of MS and waist circumference, fasting glucose, high blood pressure, BMI, psoriasis duration, high triglyceride, poor sleep quality, and high HOMA-IR, whereas high HDL levels were negatively correlated. When the effects of correlative factors were evaluated with a logistic regression analysis, only high fasting glucose, low HDL, hypertension, and waist circumference had predictive effects on the development of MS.

The present study is not without limitations. The study population is relatively small, and the study was done at only a single research center. Moreover, a relationship between the presence of MS and psoriasis severity was not established, which may seem contradictory to some literature [11,21,29]. Lastly, a relative inequality in the gender of study subjects can raise concerns regarding the validity of our findings. However, we believe that this inequality may in some sense be valuable in indicating real predominance of MS in females with severe psoriasis, although there was no significant difference in the presence of MS according to gender.

Our results are still striking enough to reach significant conclusions as follows: detection of higher MS prevalence than previously reported in Turkey [23, 24, 31], confirmation of previous predictions that the prevalence of MS will gradually increase over time, agreement with the literature that MS development increases with psoriasis duration, and significantly higher values of PSQI and HOMA-IR only in the MS group (apart from diagnostic parameters of MS) despite the absence of significant differences between the groups by age, gender or psoriasis severity. Finally, and perhaps most importantly, an inference of the present study is detection of significantly high fasting glucose and low HDL values only in females with severe psoriasis, compared to males, which may be the reason why MS was detected at a higher rate in females with psoriasis. To the best of our knowledge, this association has not been reported previously. However, controlled, broad-based and prospective studies are needed to confirm our results.

## Figures and Tables

**Table 1:** Clinical Characteristics of 112 Psoriasis Patients

Characteristic		Patients, No. (%)
Gender	M	53 (47.3)
	F	59 (52.7)
BMI (kg/m^2^)	<30	56 (50.0)
	≥30	56 (50.0)
Waist circumference (cm)	M<102, F<88	24 (21.4)
	M>102, F>88	88 (78.6)
Alcohol consumption		17 (15.2)
Smoking habit		43 (38.4)
Triglyceride (mg/dl)	<150	19 (17.0)
	≥150	93 (83.0)
HDL (mg/dl)	M<40, F<50	69 (61.6)
	M>40, F>50	43 (38.4)
Blood pressure(mmHg)	SBP<130, DBP<85	86 (76.8)
	SBP≥130, DBP≥85	26 (23.2)
Fasting glucose(mg/dl)	<110	65 (58.0)
	≥110	47 (42.0)
HOMA-IR	<2.5	41 (36.6)
	≥2.5	71 (63.4)
PASI	≤10	29 (25.9)
	>10	83 (74.1)
PSQI	<5	17 (15.2)
	≥5	95 (84.8)
Comorbidities		
Chronic lung disease		1 (0.9)
Chronic kidney disease		0 (0)
Cerebrovascular disease		0 (0)
Thyroid disease		5 (4.5)
Hypertension		26(23.2)
Diabetes mellitus		23 (20.5)

BMI = body mass index; DBP = diastolic blood pressure; F = female; HDL = high-density lipoprotein; HOMA-IR = homeostatic model assessment insulin resistance; M = male; PASI=Psoriasis Area and Severity Index; PSQI = Pittsburg Sleep Quality Index; SBP = systolic blood pressure.

**Table 2:** Clinical Characteristics of Psoriasis Patients Without (n = 36) and With (n = 76) Metabolic Syndrome: Analysis of Frequencies

Characteristic		Non-MS group^1^	MS group^1^	P^2^
Gender	M	20 (55.6)	33 (43.4)	.230
	F	16 (44.4)	43 (56.6)	
Waist circumference (cm)	M<102, F<88	19 (52.8)	5 (6.6)	.0001
	M>102, F>88	17 (47.2)	71 (93.4)	
BMI (kg/m^2^) Triglyceride (mg/dl)	≥30	11 (30.6)	45 (59.2)	.0001
	<150	14 (38.9)	5 (6.6)	.0001
	≥150	22 (61.1)	71 (93.4)	
HDL (mg/dl)	M<40, F<50	28 (22.2)	15 (80.3)	.0001
	M>40, F>50	8 (77.8)	61 (19.7)	
Fasting glucose (mg/dl)	<110	33 (91.7)	32 (42.1)	.0001
	≥110	3 (8.3)	44 (57.9)	
Blood pressure (mmHg)	SBP<130, DBP<85	35 (97.2)	51 (67.1)	.0001
	SBP≥130, DBP≥85	1 (2.8)	25 (32.9)	
PASI	≤10	12 (33.3)	17 (22.4)	.216
	>10	24 (66.7)	59 (77.6)	
PSQI	<5	9 (25.0)	8 (10.5)	.046
	≥5	27 (75.0)	68 (89.5)	
HOMA-IR	<2.5	19 (52.8)	22 (29.0)	.014
	≥2.5	17 (47.2)	54 (71.1)	
Smoking habit		14 (38.9)	29 (38.2)	.941
Alcohol consumption		5 (13.9)	12 (15.8)	.793

1 Values are no. (%) of patients.

2 Pearson’s chi-square test.

BMI = body mass index; DBP = diastolic blood pressure;F = female; HDL = high-density lipoprotein; HOMA-IR = homeostatic model assessment insulin resistance;M = male; PASI = Psoriasis Area and Severity Index; PSQI = Pittsburg Sleep Quality Index;SBP = systolic blood pressure.

**Table 3:** Clinical Characteristics of Psoriasis Patients Without (n = 36) and With (n = 76) Metabolic Syndrome: Analysis of Means

Characteristic	No-MS group^1^	MS group^1^	P
Age (years)	44.17±13.24	47.58±13.1	.202*
BMI (kg/m^2^)	28.33±4.08	31.15±4.88	.003*
Waist circumference(cm)	97.47±13.3	112.54±12.14	.0001*
Triglyceride (mg/dl)	163.81±62.47	203.68±80.24	.01*
HDL (mg/dl)	48.13±10.07	39.75±5.21	.0001*
Fasting glucose (mg/dl)	97.82±12.41	115.92±21.98	.0001*
HOMA-IR	2.39±1.06	3.48±3.19	.048ǂ
PASI	22.59±18.69	23.97±15.83	.684*
Disease duration (years)	8.76±5.78	13.09±8.99	.01ǂ
PSQI (Total)	7.94±4.72	10.01±4.78	.034*
PSSQ	1.75±0.87	1.79±0.79	.812ǂ
PSL	1.25±1.00	1.74±1.00	.018ǂ
PSDu	1.17±1.13	1.43±1.20	.266ǂ
PHSE	0.61±0.99	0.92±1.12	.159ǂ
PSDi	1.36±0.64	1.62±0.65	.052ǂ
PUSM	0.58±0.81	0.71±0.92	.480ǂ
PDSD	1.22±0.96	1.79±0.88	.003ǂ

1 Values are mean (SD).

* Independent-samples t test.

ǂ Mann-Whitney U non-parametric test

BMI = body mass index; HOMA-IR = homeostatic model assessment insulin resistance; PASI = psoriasis severity index; PDSD = Pittsburg daytime sleep dysfunction; PHSEnm = Pittsburg habitual sleep efficiency; PSDi = Pittsburg sleep disturbance; PSDu = Pittsburg sleep duration; PSL = Pittsburg sleep latency; PSQI = Pittsburg sleep quality index;PSSQ = Pittsburg subjective sleep quality; PUSM = Pittsburg use of sleeping medication.

**Table 4:** Comparison of Metabolic SyndromeParameters, by PASI and Gender1

Parameter	Gender		PASI				P^2^
			≤10 (n = 29)		>10 (n = 83)		
Waist circumference(cm)	M	<102	3	20.00%	13	34.21%	.310
		>102	12	80.00%	25	65.79%	
	F	<88	3	21.43%	5	11.11%	.325
		>88	11	78.57%	40	88.89%	
BMI (kg/m2)	M	≥30	14	26.41%	39	73.58%	
	F	≥30	15	25.42%	44	74.57%	.052
Blood pressure(mmHg)	M	SBP<130, DBP<85	12	80.00%	29	76.32%	.773
		SBP>130, DBP>85	3	20.00%	9	23.68%	
	F	SBP<130, DBP<85	12	85.71%	33	73.33%	.342
		SBP>130, DBP>85	2	14.29%	12	26.67%	
Fasting glucose(mg/dl)	M	<110	11	73.33%	19	50.00%	.123
		≥110	4	26.67%	19	50.00%	
	F	<110	13	92.86%	22	48.89%	.003
		≥110	1	7.14%	23	51.11%	
HOMA-IR	M	<2.5	7	46.67%	13	34.21%	.399
		≥2.5	8	53.33%	25	65.79%	
	F	<2.5	7	50.00%	14	31.11%	.197
		≥2.5	7	50.00%	31	68.89%	
Triglyceride (mg/dl)	M	<150	1	6.67%	3	7.89%	.879
		>150	14	93.33%	35	92.11%	
	F	<150	6	42.86%	9	20.00%	.086
		>150	8	57.14%	36	80.00%	
HDL (mg/dl)	M	<40	7	46.67%	23	60.53%	.359
		>40	8	53.33%	15	39.47%	
	F	<50	8	57.14%	38	84.44%	.031
		>50	6	42.86%	7	15.56%	
Metabolic syndrome diagnosis	M	MS (−)	5	33.33%	15	39.47%	.678
		MS (+)	10	66.67%	23	60.53%	
	F	MS (−)	7	50.00%	9	20.00%	.027
		MS (+)	7	50.00%	36	80.00%	
	Total	MS (−)	12	41.38%	24	28.92%	.216
		MS (+)	17	58.62%	59	71.08%	

1 Values are no. (%) of patients. ^2^ Pearson’s chi-square test

BMI = body mass index; HOMA-IR = homeostatic model assessment insulin resistance; MS = metabolic syndrome; PASI = Psoriasis Area and Severity Index.

**Table 5:** Pearson’s rho (and P value) Between a Diagnosis of Metabolic Syndrome (MS) and Clinical Characteristics for 112 Psoriasis Patients

MS	Disease duration	BMI	Waist circumference	Triglyceride	HDL	Blood Pressure	Fasting glucose	HOMA-IR	PASI	PSQI	Smoking habit	Alcohol consumption	
1	0.244	0.275	0.526	0.243	−0.279	0.333	0.469	0.187	0.039	0.2	−0.007	0.025	MS
	(0.01)	(0.003)	(0.0001)	(0.01)	(0.003)	(0.0001)	(0.0001)	(0.048)	(0.684)	0.034)	(0.941)	(0.796)	
	1	0.213	0.085	0.06	−0.139	0.127	0.212	−0.081	0.042	0.18	0.002	0.105	Disease
		(0.024)	(0.373)	(0.53)	(0.144)	(0.182)	(0.025)	(0.395)	(0.66)	(0.057)	(0.984)	(0.269)	duration
		1	0.447	0.096	0.031	0.219	0.04	0.103	0.012	0.053	0.042	−0.057	BMI
			(0.0001)	(0.314)	(0.743)	(0.021)	(0.675)	(0.281)	(0.902)	(0.576)	(0.659)	(0.554)	
			1	0.085	−0.022	0.184	0.135	0.052	−0.017	0.2	−0.035	−0.082	Waist
				(0.373)	(0.814)	(0.052)	(0.155)	(0.589)	(0.86)	(0.034)	(0.713)	(0.388)	circumference
				1	−0.063	0.152	0.18	0.235	0.158	0.152	0.043	0.046	Triglyceride
					(0.512)	(0.109)	(0.058)	(0.013)	(0.096)	(0.11)	(0.651)	(0.628)	
					1	−0.118	−0.154	0.031	0.204	0.122	0.086	−0.069	HDL
						(0.216)	(0.106)	(0.746)	(0.031)	(0.202)	(0.369)	(0.468)	
						1	0.004	0.206	0.15	−0.031	0.044	0.062	Blood
							(0.968)	(0.03)	(0.115)	(0.746)	(0.643)	(0.515)	pressure
							1	0.199	0.187	0.265	−0.002	−0.057	Fasting
								(0.035)	0.048)	(0.005)	(0.986)	(0.549)	glucose
								1	−0.009	0.013	0.18	0.041	HOMA-IR
									(0.926)	(0.893)	(0.057)	(0.671)	
									1	0.298	−0.046	0.073	PASI
										(0.001)	(0.632)	(0.443)	
										1	0.004	0.042	PSQI
											(0.967)	(0.662)	
											1	0.434	Smoking
												(0.0001)	habit
												1	Alcohol
													consumption

BMI = body mass index; HOMA-IR = homeostatic model assessment insulin resistance; MS = metabolic syndrome; PASI = Psoriasis Area and Severity Index; PSQI = Pittsburgh Sleep Quality Index.

**Table 6:** Predictivity of Correlative Parameters on the Development of Metabolic Syndrome, by Logistic Regression

Parameter	P	OR (95% CI)
Disease duration (years)	.485	1.04 (0.93 to 1.16)
BMI (kg/m^2^)	.471	1.06 (0.90 to 1.26)
Waist circumference (cm)	.037	1.07 (1.00 to 1.14)
Fasting glucose	.004	1.09 (1.03 to 1.15)
HOMA-IR	.65	0.89 (0.54 to 1.47)
Low HDL	.005	0.84 (0.75 to 0.95)
Triglyceride	.282	1.01 (0.99 to 1.03)
Hypertension	.02	0.04 (0to 0.60)
PASI	.223	2.76 (0.54 to 14.08)
PSQI	.918	0.91 (0.14 to 5.91)

BMI = body mass index; CI = confidence interval; HOMA-IR = homeostatic model assessment insulin resistance; OR = odds ratio; PASI = Psoriasis Area and Severity Index; PSQI = Pittsburg Sleep Quality Index.
